# Developing a Core Outcome Set for blood purification in hypertriglyceridemic acute pancreatitis: a Delphi study protocol

**DOI:** 10.3389/fmed.2025.1645585

**Published:** 2025-11-10

**Authors:** Shengteng Guo, Haoran Zhu, Rongzhen Zhao, Qinghai Guan, Juan Wu, Qinghua Wang

**Affiliations:** 1 School of Nursing (School of Gerontology), Binzhou Medical University, Binzhou, Shandong, China; 2 Department of Hepatobiliary and Pancreatic Surgery, Binzhou Medical University Hospital, Binzhou, Shandong, China

**Keywords:** hypertriglyceridemia, acute pancreatitis, blood purification, Core Outcome Set, Delphi

## Abstract

**Background:**

Early initiation of lipid-lowering therapy is crucial for controlling disease progression and preventing recurrent acute pancreatitis in patients with hypertriglyceridemic acute pancreatitis (HTGP). As a clinically common intervention for HTGP, blood purification enables rapid clearance of serum triglycerides. However, the current absence of a standardized outcome measurement system for blood purification in HTGP hinders systematic therapeutic evaluation by clinicians and further impedes the integration and comparison of research findings. Consequently, this study aims to develop a Core Outcome Set (COS) for blood purification in HTGP, standardizing the assessment of treatment efficacy and enhancing comparability across similar studies.

**Methods:**

The COS will be developed in accordance with the Core Outcome Measures in Effectiveness Trials (COMET) Handbook, Core Outcome Set-STAndardised Protocol (COS-STAP), Core Outcome Set-STAndards for Development (COS-STAD), and Core Outcome Set-STAndards for Reporting (COS-STAR). First, research team members and a stakeholder panel will be recruited. Outcome indicators relevant to stakeholders will be identified through systematic reviews and interviews. The stakeholder panel and domain experts will then participate in a two-round Policy Delphi process followed by a consensus meeting to discuss and score potential outcomes, ultimately determining the final COS. The finalized COS will be disseminated via a peer-reviewed publication and a checklist to facilitate implementation and promotion.

**Discussion:**

The COS for blood purification in HTGP will establish a standardized, evidence-based outcome measurement framework. This structured approach will: (1) Enable comprehensive therapeutic evaluation by clinical teams, (2) Enhance precision in disease progression control, (3) Reduce heterogeneity across comparable studies, and may ultimately inform global treatment practices for HTGP.

**Standardized registration statement:**

This study has been prospectively registered with the Core Outcome Measures in Effectiveness Trials (COMET) initiative (ID: 3231; Accessible at: https://www.comet-initiative.org/Studies/Details/3231) and the China Clinical Trial Core Outcome Sets Research Center (ChiCOS) (ID: CHICOS2024000024; Accessible at: https://www.chicos.org.cn/home).

## 1 Introduction

Acute pancreatitis (AP) is a prevalent gastrointestinal emergency characterized by localized and systemic inflammatory responses, with potential progression to severe acute pancreatitis (SAP)—a condition associated with high mortality. In China, hyperlipidemia has superseded alcohol as the second leading etiology of AP, primarily driven by significant elevation of serum triglycerides. Hence, hyperlipidemic acute pancreatitis may be alternatively termed hypertriglyceridemic acute pancreatitis (HTGP) (1). In Western countries, hypertriglyceridemia represents the most prevalent etiology of AP after biliary diseases and alcohol exposure. Notably, AP cases conjointly triggered by alcohol and hyperlipidemic diets exceed the incidence of idiopathic AP (2–4). Current epidemiological data indicate an HTGP prevalence rate of 14%, demonstrating an upward trajectory (5). Compared to AP of other etiologies, HTGP exhibits significantly higher recurrence rates and greater propensity to progress to HTG-SAP (6). Critical evidence indicates serum triglyceride (TG) levels directly modulate disease progression: each 1.13 mmol/L increment elevates AP risk by 4% (7, 8). Consequently, early initiation of lipid-lowering therapy is imperative for mitigating disease advancement and preventing recurrent AP in HTGP patients (7).

Conventional lipid-lowering therapy for HTGP encompasses insulin therapy, oral pharmacologic agents, and dietary modification. For patients refractory to these measures, blood purification serves as an essential therapeutic modality to achieve rapid triglyceride clearance, establishing its role as a clinically common intervention in HTGP management. Blood purification techniques refer to therapeutic methods that extract blood from the body through extracorporeal circulation devices to remove harmful substances or substitute organ functions, which primarily includes continuous renal replacement therapy (CRRT), hemoperfusion (HP), plasma exchange (PE), and immunoadsorption. Clinical evidence has shown that implementing blood purification within 72 h can rapidly reduce serum TG levels, significantly alleviate patient symptoms, effectively mitigate systemic inflammatory responses and decrease complication rates. Simultaneously, it improves patient survival rates and reduces the recurrence risk of AP (9–11).

Core Outcome Set (COS) is defined as a consensus-based standardized set of measurable and reportable outcomes, commonly used to guide outcome selection in clinical trials, care practices, and systematic reviews (12). COS serves to reduce selective reporting of study outcomes and enhance comparability and relevance across similar studies (13). Despite the existence of COS for pharmacological clinical trials and pain control in acute pancreatitis, a dedicated and standardized COS for blood purification in HTGP remains lacking (14, 15).

Current outcome indicators for evaluating blood purification therapy in HTGP include mortality rate, complication incidence, length of hospital stay, treatment costs, as well as laboratory parameters such as TG, C-reactive protein (CRP), and interleukin-7 (IL-7). Researchers can utilize these indicators to assess treatment efficacy and infer patient prognosis (9, 16, 17). However, a literature review has revealed that different studies have chosen varying outcome indicators, leading to discrepancies across research findings. Furthermore, due to the lack of standardized outcome measures, some studies fail to include all indicators that are effective for HTGP blood purification therapy. This significantly limits the comparability of findings and the integration of evidence, thereby impeding clinical decision-making and international collaboration (18, 19). Additionally, outcome reporting bias exacerbates the challenges faced by healthcare decision-makers (20).

Although medications and blood purification show similarities in reported outcomes for lipid-lowering effects, different treatment modalities imply variations in reported outcomes. This study aims to develop a COS specifically for blood purification therapy in HTGP through systematic review, Delphi, and consensus conference, thereby addressing inconsistencies in outcome reporting and improving the comparability of international research. The applicability of this COS extends to randomized controlled trials and observational studies. Its implementation by clinicians, researchers, nurses, and healthcare policymakers in the contexts of multicenter research and clinical practice is poised to advance the management and measurement of HTGP.

## 2 Methods and analysis

### 2.1 Design

**FIGURE 1:**
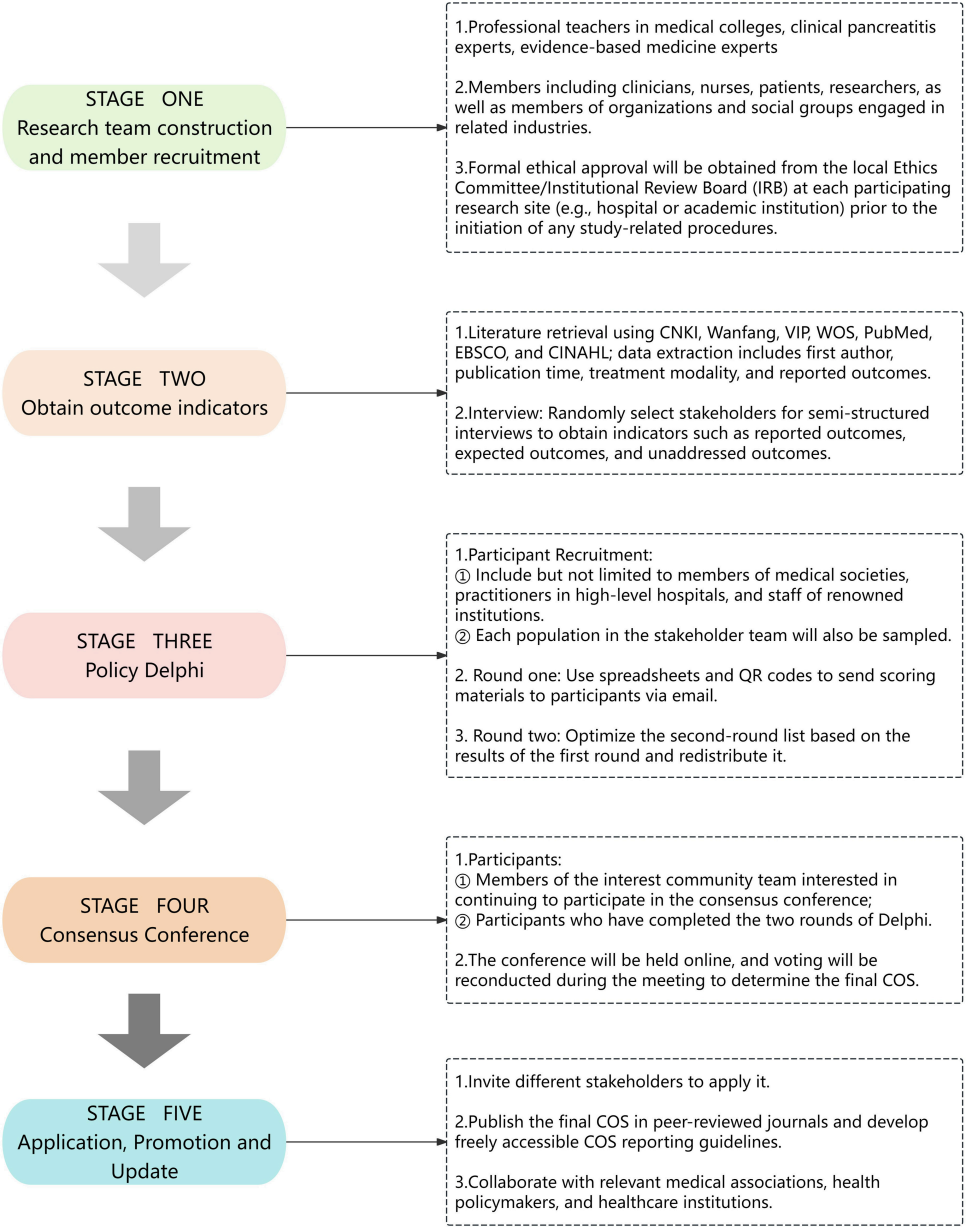
Flowchart of study design. COS, Core Outcome Set.

The COS for blood purification therapy in HTGP was designed based on the Core Outcome Measures in Effectiveness Trials (COMET) Handbook, Core Outcome Set-STAndardised Protocol (COS-STAP), Development (COS-STAD), and Reporting (COS-STAR) guidelines (12, 13, 20, 21). The study comprises five components: (a) Construction of the research team and member recruitment; (b) Acquisition of research indicators through systematic literature retrieval and interviews with stakeholders (including physicians, nurses, patients); (c) Identification of critical outcomes via a policy Delphi survey involving experts and stakeholders; (d) Conducting a consensus conference for finalization of outcome indicators; (e) Implementation, updating, and dissemination of the COS in healthcare settings (Figure 1).

### 2.2 Stage 1: research team construction and recruitment

The research team comprises professional faculty members from medical schools (10%), clinical experts (including physicians, nurses, and technicians, 50%), evidence-based medicine (EBM) specialists (10%), and graduate research assistants specializing in pancreatology (30%), totaling 20 members. Among them, faculty members have at least 10 years of teaching experience and engage in clinical practice at least once annually. Clinical experts possess over 20 years of clinical experience, are currently engaged in research on HTGP, and hold leadership positions as department heads. Additionally, both faculty and clinical experts have published at least one research article on AP annually between 2023 and 2025. Evidence-based medicine experts are required to provide guidance on research design, monitor the research process, and adjust deviations in protocol implementation based on evidence. The research team is responsible for conducting literature reviews, administering interviews, implementing the Delphi process, and organizing consensus conferences.

We also intend to establish a stakeholder team for blood purification therapy in HTGP, comprising clinicians, nurses, patients, researchers, and members from related industries, organizations, and social groups. The selection criteria are as follows: (1) Individuals who have performed blood purification therapy or provided post-treatment care within the past 3 years; (2) Individuals diagnosed with HTGP who have received blood purification therapy within the past 3 years, or their family members; (3) Personnel engaged in research on blood purification procedures, medical device development, social support. Stakeholders will be recruited through ChiCOS and medical social platforms. A total of 50 participants are planned to be recruited, with an anticipated attrition rate of 20%. The study primarily targets individuals proficient in both English and Chinese. The research team will conduct a thorough screening of potential participants, including verification of professional qualifications and medical visit records, to ensure compliance with the study’s eligibility criteria. The finally recruited stakeholders will be categorized by country or region, with efforts to include regions with varying AP burden levels (22). This aims to ensure the diversity of the COS and ultimately enhance its global applicability.

### 2.3 Stage 2: screening of outcome measures

#### 2.3.1 Systematic review

A systematic search and review of literature related to blood purification therapy in HTGP will be conducted to identify reported outcome measures. A detailed protocol for the systematic review has been registered in PROSPERO (CRD420251062542). A literature search will be conducted in CNKI, WanFang, VIP, Web of Science (WOS), PubMed, EBSCO, and CINAHL, spanning from database inception to June 2025. The search will integrate subject terms with Medical Subject Headings (MeSH) terms, including keywords such as “acute pancreatitis,” “hyperlipidemia,” “hypertriglyceridemia,” “blood purification,” “blood lipids,” “prognosis,” “outcome,” and “standards.” Inclusion and exclusion criteria for the literature are presented in Table 1.

**TABLE 1:** Inclusion and exclusion criteria for systematic review.

Criteria	Description	
Inclusion	Patient	Patients with a confirmed diagnosis of HTGP: (1) Diagnostic criteria for pancreatitis (31): ① Acute, sudden-onset, persistent, severe epigastric pain radiating to the back; ② Serum amylase and/or lipase levels ≥ 3 times the upper limit of the reference range; ③ Contrast-enhanced CT or MRI showing typical imaging changes of AP: pancreatic edema or peripancreatic exudative effusion. Diagnosis of AP is established if two of the three criteria are met. (2) Diagnostic criteria for hyperlipidemia (6): Serum TG ≥ 11.3 mmol/L, or TG between 5.65 and 11.3 mmol/L with chylous appearance of the serum.
	Intervention	Any form of blood purification therapy, including but not limited to CRRT, PE, HP, and immunoadsorption.
	Control	Including but not limited to lipid-lowering medications (statins, fibrates, niacin), insulin, and conventional therapy.
	Type	Randomized controlled trials, qualitative studies, quantitative studies, mixed methods studies, and systematic reviews related to blood purification therapy for HTGP.
Exclusion	–	Animal studies, conference abstracts, studies with unavailable full-text, or incomplete research data.

HTGP, hypertriglyceridemic acute pancreatitis; CRRT, continuous renal replacement therapy; PE, plasma exchange; HP, hemoperfusion.

#### 2.3.2 Interview

The recruited stakeholder team will be categorized into hospital-related groups, patient-related groups, and society-related groups. Stakeholders from each category will be randomly selected for semi-structured interviews, with the interview duration limited to 20–30 min. The number of interviewees is not restricted, and data collection will continue until data saturation is achieved. All interviewees are required to sign an informed consent form, and the Standards for Reporting Qualitative Research (SRQR) will be adopted as the reporting guideline (23). The interviews aim to elicit outcomes of concern regarding blood purification therapy for HTGP from diverse stakeholder perspectives, ensuring the diversity and representativeness of outcome measures. The interview guides for different groups are presented in Table 2.

**TABLE 2:** Interview guides for different stakeholder groups.

Groups	Interview guides
Hospital-related	1. What specific work related to blood purification for HTGP are you currently involved in? 2. Which outcomes of the blood purification treatment process do you pay particular attention to in your work? 3. Besides these, what other aspects do you think should be considered as outcomes worthy of attention? 4. Is there anything else you would like to add?
Patient-related	1. Have you or your family members ever received blood purification therapy? How was the experience? 2. During the blood purification treatment, what aspects were you particularly concerned about? 3. If you or your family members were to receive blood purification therapy again, what other treatment-related aspects do you think you would pay attention to? 4. Is there anything else you would like to add?
Society-related	1. What specific work related to blood purification are you currently engaged in? 2. Based on your professional experience, which outcome indicators do you think require attention in blood purification therapy? 3. If you were to receive blood purification treatment, do you believe there are other outcome indicators that deserve attention? 4. Is there anything else you would like to add?

HTGP, hypertriglyceridemic acute pancreatitis.

#### 2.3.3 Data extraction and processing

The systematic review data will be extracted independently by two researchers, including first author, publication year, treatment modality, and reported outcomes. Following data extraction, both researchers will jointly perform data entry. In cases of discrepancies regarding reported outcomes, evidence-based medicine experts and clinical specialists will be consulted for final determination. Interview processes will be documented through audio recording and handwritten notes. Interviewers must verify the extracted data with stakeholders within 24 h after the interview, including reported outcomes, anticipated outcomes, and unaddressed outcomes, to ensure data accuracy and confirm no new information emerges.

The outcome measures obtained from systematic reviews and interviews will be integrated to construct a COS. Duplicate or highly similar outcomes will be systematically consolidated into single entries. The COS shall then be structured according to established medical classification systems to streamline the Delphi process (24). The sequence and quantity of outcomes will be optimized to mitigate potential biases during the Delphi study (25). All data processing will be exclusively conducted by the research team to ensure confidentiality without third-party involvement. Any discrepancies will be resolved through consultation with senior team members.

### 2.4 Stage 3: policy Delphi

Unlike the traditional Delphi method, Policy Delphi participants have known identities and do not pursue opinion consensus, but rather aim to reveal the breadth and diversity of perspectives (26). This approach encourages participants to express divergent views, solicits opinions from multi-stakeholder groups, and ensures the pluralism of research findings. The process will be conducted online over two rounds, with each round anonymously determining key outcomes and priority rankings. A consensus on the COS items for blood purification therapy in HTGP will be reached ultimately.

Delphi participants will be recruited through multiple channels to ensure the diversity and representativeness of outcomes. First, renowned experts in the field will be identified via literature searches and online research, including but not limited to members of medical societies, practitioners from top-tier hospitals, and faculty from prestigious institutions. These experts will derive from diverse settings, bringing experience across research, prevention, and primary care domains to represent multi-level perspectives. Invitations will be extended to first or corresponding authors who have published at least five peer-reviewed articles on blood purification and HTGP, a criterion designed to ensure the translatability of research findings. In addition, stakeholders from each subgroup of the stakeholder team will be sampled to participate in the Delphi process. Through this comprehensive Delphi approach, we aim to ensure not only the clinical relevance and diversity of the final COS, but also the sustainability of research findings translation and updating.

If participant recruitment falls short of the target (*n* = 50), the research team will extend the recruitment period. Each round will last for 3 weeks to ensure participants have sufficient time to respond. To ensure timely replies, the research team will send reminder emails to non-responding participants after the end of the second week. There will be a 2-week interval between rounds for data statistical analysis and preparation for subsequent steps. The research team will conduct a rigorous check of Round 1 data and refine the outcome list based on participants’ responses. This two-round Delphi with feedback has been validated in other COS studies and is a standard practice for developing healthcare consensus (27).

#### 2.4.1 Round 1

The importance of each outcome measure will be evaluated using a nine-point Likert scale, where 1–2 indicates “very unimportant,” 3–4 “unimportant,” 5 “neutral,” 6–7 “important but not critical,” and 8–9 “critical” (28). The outcome measures included in the list are derived from both systematic reviews and interview results, each accompanied by a detailed description. Participants are allowed to provide written rationales for their ratings in text form. Additionally, they are encouraged to supplement the list by identifying “critical” outcome measures and justifying their choices, which ensures the completeness of research findings.

The list will be sent to participants via email in the form of a spreadsheet and a QR code. Participants can review the outcome list and its explanations in the spreadsheet and complete preliminary entries. Final submission will be done by scanning the QR code, enabling real-time data capture to ensure prompt responses. All items are set as “mandatory fields” to prevent omissions and ensure completeness of responses.

#### 2.4.2 Round 2

The second-round list will be optimized based on first-round results. An outcome measure will not be included in the second round if > 70% of participants rate it ≤ 4 points or < 15% rate it ≥ 8 points. Newly proposed outcomes from the first round and those failing to reach consensus will be added to the second-round list for re-rating, sent via email using the same method as the first round. Participants who did not complete the first round will not be invited to the second round. Second-round participants will receive first-round feedback, including mean scores for each outcome, exclusion status, newly added items, and their own first-round ratings.

#### 2.4.3 Data management

The data from both rounds will be managed and analyzed using online survey software. After the survey concludes, the research team will download data to local storage and anonymize all information to protect participants’ privacy. Statistical software will be employed for analysis, using conventional methods such as chi-square tests, *t*-tests, means, and word frequency charts to further explore support rates and importance of outcome measures. The final data from both rounds will be returned to the Delphi participants. Any indicators on which disagreement persists will be included in a consensus meeting. If participant attrition occurs in either round, sensitivity analysis will be conducted to assess whether sample size affects final conclusions (29).

### 2.5 Stage 4: consensus conference

The consensus conference aims to bring together diverse stakeholders, particularly patients, to achieve consensus (27). The consensus meeting will convene approximately 15–20 key participants, comprising clinical experts, nursing representatives, methodological specialists, and patient representatives who have completed both Delphi rounds. Participants will be selected based on a comprehensive consideration of their professional background, geographical distribution, gender, level of disease burden, and healthcare setting to ensure diverse perspectives. Furthermore, priority will be given to those who have completed the entire Delphi process to guarantee an in-depth understanding of the candidate outcome measures and to uphold the professionalism of the consensus results.

To ensure inclusivity across geographical locations, the consensus meeting will be held via an online video platform. The definitive list of core outcomes will be finalized based on pre-defined criteria (Table 3). To promote informed and efficient deliberations, a comprehensive package—including the final set of candidate outcomes, their respective Delphi survey scores, and response distributions—will be disseminated to all participants one week preceding the meeting. An impartial methodological expert will chair the proceedings to maintain a structured discussion. Each outcome indicator will be addressed in sequence. Deliberations will extend to all outcomes, irrespective of their prior consensus status in the Delphi study, with active participation encouraged to furnish additional clinical rationale or supporting evidence. Particular emphasis will be placed on the re-evaluation of outcomes that demonstrated ambiguity or significant dissent during the Delphi process, to reach a definitive conclusion regarding their inclusion in the final COS.

**TABLE 3:** Consensus criteria for outcome list.

Classification	Description	Definition
Consensus	This outcome indicator should be included in the final COS	More than 70% of participants rated the outcome as ≥ 8 points, or less than 15% rated it as ≤ 4 points
Not- Consensus	This outcome indicator should not be included in the final COS	More than 70% of participants rated the outcome as ≤ 4 points, or less than 15% rated it as ≥ 8 points
Ambiguous	Uncertain whether it should be included in the COS	More than 50% of participants rated the outcome as five points

COS, Core Outcome Set. Outcomes identified as ambiguous will be designated for in-depth discussion and re-evaluation to determine their inclusion in the COS.

Upon conclusion of the deliberations, an online anonymous voting procedure will be undertaken for every outcome indicator. This will be followed by a real-time tallying of votes and the immediate announcement of a preliminary COS list. Prior to formal adjournment, participants will be afforded an opportunity to briefly reconsider and summarize their positions in response to the preceding discussion. The panel’s final task will be to collectively confirm the practical feasibility, ease of assessment, and generalizability of the endorsed outcomes across a range of healthcare environments.

All voting data will be collected through a professional online survey platform to ensure complete anonymity. The research team will only have access to the aggregated results and cannot trace any individual responses. The entire meeting will be audio-recorded for internal records only; all personally identifiable information will be removed from the transcribed transcripts. All research data will be stored on an encrypted, password-protected server, accessible exclusively to core research members. Additionally, all participants are required to sign a confidentiality agreement to prevent disclosure of discussion content and interim proceedings.

Following the consensus meeting, the research team will distribute the final COS list, meeting minutes, and a summary of voting results to all participants via email within 2 weeks.

### 2.6 Stage 5: application, promotion and update

The COS for blood purification therapy in HTGP will be applied by inviting diverse stakeholders across different countries, regions, socioeconomic conditions, and healthcare settings to validate its effectiveness and generalizability. The research team will prioritize dissemination of findings, including publishing the final COS in peer-reviewed journals, developing freely accessible COS tables, and engaging interested stakeholders in promotion efforts. Collaboration will continue with relevant medical associations, health policymakers, and healthcare institutions for COS implementation and updating, including systematic reviews of emerging evidence and may involve initiating new Delphi surveys or consensus meetings as necessary, thereby ensuring its continued alignment with the needs of both clinical practice and research.

## 3 Expected outcomes

This study develops a COS for blood purification therapy in HTGP through systematic review, interviews, Delphi surveys, and consensus conferences, aiming to provide a standardized tool for evaluating the efficacy of blood purification. This tool is intended to be scientific, inclusive, and generalizable, applicable across different countries, healthcare settings, and regions with varying pancreatitis burdens.

The COS will bridge existing research gaps by standardizing outcomes prioritized in blood purification therapy studies. The included measures will offer distinct insights to physicians, nurses, and health policymakers, enabling evidence-based management of patient progression and informed health policy development. Its multi-stakeholder design ensures alignment with clinical practice realities and research needs, establishing a robust framework for future HTGP investigations.

The COS for blood purification therapy in HTGP will ultimately be presented in a peer-reviewed journal publication. Concurrently, the research team will provide a standardized reporting framework to enhance implementation and promotion efficiency.

## 4 Discussion

This study aims to develop a COS for blood purification in HTGP through systematic, evidence-based methodology. The research team has designed the protocol adhering to EBM principles, with formal consultation of EBM methodologists to enhance methodological robustness and framework stability. Further rigor was achieved by compliance with established reporting guidelines (12, 13, 20, 21). As a standardized implementation tool, the COS provides an evidence-derived minimum outcome measurement suite. Its implementation will offer researchers standardized endpoints, harmonize outcome reporting priorities, reduce heterogeneity across studies, improve comparative validity, and ultimately shape global practice standards for lipid-lowering therapy in HTGP.

Stakeholder panel selection extends beyond conventional inclusion of patients and clinicians to incorporate representatives from relevant industries and patient advocacy organizations. This multinational cohort exhibits diverse clinical profiles, healthcare access levels, and pancreatitis burden, enabling comprehensive outcome identification that ensures COS global applicability across varied care settings. Geographically stratified experts further validate the COS through structured consultations, collectively establishing a scientifically rigorous framework with cross-context adaptability.

The final COS determination will be achieved through a structured consensus conference engaging both the stakeholder panel and Delphi study participants. Experts stratified by experience level will conduct rigorous deliberations to ensure terminological precision, conceptual clarity, and implementation fidelity of outcome measures. Parallel focus groups with distinct stakeholder cohorts will evaluate context-specific applicability and scalability pathways. Post-conference, stakeholders will be formally designated as dissemination ambassadors to enhance adoption efficiency. Through iterative real-world validation and biennial updates, this initiative will generate policy-grade evidence for health authorities, ultimately driving guideline development for blood purification and lipid management in HTGP, while informing healthcare policy reform.

This study acknowledges inherent limitations regarding linguistic inclusivity. Participation was restricted to individuals proficient in English, potentially introducing selection bias favoring perspectives from Anglophone regions. Furthermore, dissemination outputs will be available only in English and Chinese, which may impede accessibility in non-target linguistic zones. Translation inaccuracies during local adaptation could also compromise conceptual fidelity. To mitigate these constraints, the research team will commission ISO-certified medical translators to produce culturally validated versions of the COS following its finalization, supplemented by cognitive debriefing with local clinicians to ensure terminological accuracy. In subsequent research, the research team will need to further refine the measurement standards for measurable data. The development of measurement tools and standards will follow the Consensus-based Standards for the Selection of Health Measurement INstruments (COSMIN) methodology framework to facilitate more precise measurement of outcome indicators (30).

In summary, the development of a standardized and evidence-based COS for blood purification therapy in HTGP provides uniform and scientific outcome measures for treatment. This will assist healthcare teams in more precisely monitoring disease progression and reduce heterogeneity across similar studies. The final COS is expected to be incorporated into clinical guidelines or policy-making, potentially influencing global treatment practices for HTGP.
